# Characterization of human epithelial resident memory regulatory T cells

**DOI:** 10.3389/fimmu.2022.962167

**Published:** 2022-08-19

**Authors:** Takuya Sato, Youichi Ogawa, Kazunori Yokoi, Yuka Nagasaka, Aoha Ishikawa, Ichiro Shiokawa, Manao Kinoshita, Rei Watanabe, Shinji Shimada, Atsushi Tanaka, Akira Momosawa, Tatsuyoshi Kawamura

**Affiliations:** ^1^ Department of Dermatology, Faculty of Medicine, University of Yamanashi, Yamanashi, Japan; ^2^ Department of Dermatology, Course of Integrated Medicine, Graduate School of Medicine, Osaka University, Osaka, Japan; ^3^ Department of Plastic Surgery, Faculty of Medicine, University of Yamanashi, Yamanashi, Japan; ^4^ Experimental Immunology, Immunology Frontier Research Center, Osaka University, Osaka, Japan

**Keywords:** resident memory CD4 + T cell, regulatory (Treg) cell, epithelia, human, demethylation, foxp3, CD103

## Abstract

Human resident memory regulatory T cells (Tregs) exist in the normal, noninflamed skin. Except one, all previous studies analyzed skin Tregs using full-thickness human skin. Considering that thick dermis contains more Tregs than thin epidermis, the current understanding of skin Tregs might be biased toward dermal Tregs. Therefore, we sought to determine the phenotype and function of human epidermal and epithelial Tregs. Human epidermis and epithelium were allowed to float on a medium without adding any exogenous cytokines and stimulations for two days and then emigrants from the explants were analyzed. Foxp3 was selectively expressed in CD4^+^CD103^−^ T cells in the various human epithelia, as it is highly demethylated. CD4^+^CD103^−^Foxp3^+^ cells suppressed proliferation of other resident memory T cells. The generation and maintenance of epithelial Tregs were independent of hair density and Langerhans cells. Collectively, immune-suppressive CD4^+^CD103^−^Foxp3^+^ Tregs are present in the normal, noninflamed human epidermis and mucosal epithelia.

## Introduction

The human skin is composed of three layers, namely, epidermis, dermis, and subcutaneous tissue. Epidermis is the outermost layer of the skin. Meanwhile, epithelium is the tissue that covers the internal and external surfaces of the body. In this context, the epidermis is a type of epithelial tissue. The dermis is basically made up of the structural protein known as collagen. It is much thicker than the epidermis and contains a variety of CD45^+^ immune cells that are derived from hematopoietic lineage cells (HLCs) (e.g., resident memory T (T_RM_) cells, dendritic cells, macrophages, natural killer cells, and mast cells). Meanwhile, the epidermis functions primarily as a barrier against various external assailants. It is believed to be primarily composed of a majority of CD45^−^ keratinocytes with CD45^−^ melanocytes, Merkel cells, and CD45^+^ Langerhans cells (LCs) as minor components. The low number of CD45^+^ HLC-derived immune cells in the epidermis resulted in the concept that the epidermis is immunologically undynamic. Therefore, a recent finding on the presence of human T_RM_ cells in the epidermis was a milestone (1).

Like human T_RM_ cells, human resident memory regulatory T cells (Tregs) exist in peripheral tissues, including the skin, under normal and noninflamed conditions (2–8). Interestingly, CD45RO^+^ antigen-experienced memory Tregs colonize even in fetal skin (6, 8). Foxp3-expressing skin Tregs are abundant in hair follicles and at the dermal−epidermal junction, accounting for approximately 10% of skin CD3^+^ T cells in the steady state condition (2, 5, 6). Experiments using murine full-thickness skin have revealed that murine skin Tregs mediate tolerance to commensal microbes (9), facilitate skin wound healing (10) and epithelial stem cell differentiation (11, 12), and inhibit profibrotic immune responses (13). However, the functions of human skin Tregs have been largely unknown, which is possibly due to the limited availability of human skin.

Proliferation of human dermal Tregs is mediated by dermal fibroblasts and IL-15 through cell−cell contact without antigen presentation and costimulation exerting their suppressive function also through cell−cell contact, independent of IL-10 and TGF-β production (2). On the other hand, proliferation of human epidermal Tregs is mediated by LCs, the epidermal and epithelial antigen-presenting cells, indicating LCs’ tolerogenic features in the steady state. These epidermal Tregs suppress the proliferation of autologous other-skin T_RM_ cells *via* cell−cell contact, dependent on MHC-II and CD80/CD86, suggesting the involvement of antigen presentation. Interestingly, the percentage of Foxp3^+^ and Ki-67^+^ cells in CD3^+^ cells is significantly higher in the epidermis than the dermis, suggesting the active *in situ* proliferation of human epidermal Tregs (5).

These previous elegant works elucidated the existence of immune-suppressive resident memory Tregs in the normal and noninflamed human skin (2–8). However, all previous studies analyzed skin Tregs using full-thickness human skin, except for one study (5). Considering that thick dermis contains more T_RM_ cells than thin epidermis (14, 15), the current understanding of skin Tregs might be biased toward dermal Tregs. Therefore, this study focused on epidermal and epithelial Tregs. In addition, human epidermal and dermal T_RM_ cells express CD69, a T_RM_ marker, further being segregated into CD103^+^ and CD103^−^ cells (1, 15, 16). Thus, a differential Foxp3 expression and function between CD103^+^ and CD103^−^ cells were also examined.

## Materials and methods

### Sources of tissues

Healthy, noninflamed human samples were obtained from routinely discarded tissue following plastic and gynecological surgeries. Unless otherwise noted, healthy and noninflamed human skin from female mamma was used in the experiments. All lesional skin samples of treatment-naive atopic dermatitis (AD) and psoriasis were taken from male patients and trunk.

### Tissue processing

RPMI 1640 (Invitrogen Life Technologies, Carlsbad, CA) containing 10% FBS (Biowest, Nuaillé, France) and Anti-Anti (1:100; Gibco, Dublin, Ireland) without additional exogenous cytokines was utilized as a culture medium. This study analyzed emigrants from the epidermis and mucosal epithelium recovered *via* spontaneous migration method. In the indicated experiment, samples were enzymatically digested. In the spontaneous migration method, the skin or mucosa was washed with sterile cold PBS (Gibco, Dublin, Ireland) immediately after surgery at day 0. To obtain epidermal or epithelial sheets, the subcutaneous fat and deep dermis or lamina propria were thoroughly removed using a pair of scissors, respectively. The parts of skin composed of the epidermis and upper dermis or mucosa composed of epithelium and upper lamina propria were cut into approximately 20 × 10 mm square pieces and then incubated with Dispase II (2.5 mg/mL for mamma, scrotum, and glans penis, 1.5 mg/mL for urethra, and 1.25 mg/mL for vagina; Roche Diagnostics, Indianapolis, IN) dissolved with PBS (Gibco, Dublin, Ireland) overnight at 4°C. In the enzymatically digestion method, the epidermal and epithelial sheets were incubated with collagenase type IV (200 U/mL; Worthington, Lakewood, NJ) for 30 min at 37°C at day 1. After incubation, the sheets were divided into small pieces using a pair of forceps. To generate single-cell suspensions, these pieces were aspirated with a 50-cc syringe up and down 5 times. This was then filtered thrice through a sterile mesh, with subsequent analysis on the same day (day 1). To obtain emigrants, the epidermal or epithelial sheets were floated in the culture medium for 2 days at 37°C. At day 3, these emigrants were analyzed.

### Flow cytometry

Following anti-human monoclonal antibodies (mAbs) were used in the study: anti-CD3 (clone: HIT3a), anti-CD4 (clone: OKT-4), anti-CD8a (clone: HIT8a), anti-CD25 (clone: M-A251), anti-CD27 (clone: M-A271), anti-CD28 (clone: 28.2), anti-CD39 (clone: A1), anti-CD69 (clone: FN50), anti-CD103 (clone: Ber-ACT8), anti-CD45RA (clone: HI100), anti-CD45RO (clone: UCHL1), anti-CD62L (clone: DREG-56), anti-CD122 (clone: TU27), anti-CD127 (clone: A019D5), anti-CD132 (clone: TUG-H4), anti-CD226 (clone: 11A8), anti-CCR7 (clone: G043H7), anti-CLA (clone: HECA-452), anti-CCR4 (clone: L291H4), anti-CTLA-4 (clone: BNI3), anti-PD-1 (clone: EH12.2H7), anti-IFN-γ (clone: B27), anti-IL-4 (clone: 8D4-8), anti-IL-17A (clone: BL1-68), and anti-TNF-α (clone: MAb-11); these mAbs were purchased from BioLegend (San Diego, CA). Anti-CD1a mAbs (clone: HI149), and anti-TGF-β1 (clone: TW4-9E7) were purchased from BD Bioscience (Franklin Lakes, NJ). Anti-Foxp3 (clone: PCH101), anti-ICOS (clone: ISA-3), and anti-TIGIT (clone: MBSA43) were purchased from eBioscience™ (San Diego, CA). Emigrants or enzymatically digested single-cell suspensions were incubated with mAbs for 30 min at 4°C and then washed twice in staining buffer and examined by FACS Caliber (BD Biosciences, Franklin Lakes, NJ). Dead cells were detected as propidium iodide-positive cells (Sigma-Aldrich, St. Louis, MO). For cytokine expression, dead cells were eliminated from epidermal emigrants using the Dead Cell Removal Kit from Miltenyi Biotec GmbH (Bergisch Gladbach, Germany). Cells were cultured at a density of 5 × 10^5^ per 48-well culture plate with 500 μL of culture medium supplemented with eBioscience Cell Stimulation Cocktail (500x) from Invitrogen (Carlsbad, CA) and eBioscience Protein Transport Inhibitor Cocktail (500x) from Invitrogen (Carlsbad, CA) for 6 h at 37°C. For intracellular staining, surface antigens were stained as described above, followed by cell fixation and permeabilization using eBioscience™ Foxp3/Transcription Factor Staining Buffer Set (Invitrogen™, Carlsbad, CA) according to the manufacturer’s instructions. Cells were then incubated with anti-Foxp3 mAbs together with mAbs for cytokines for 30 min at 4°C and washed twice in the buffer. Data were analyzed using the FlowJo software (FlowJo, LLC, Ashland, OR).

### Immunofluorescence

Healthy, noninflamed human skin samples were fixed for 24 h in 4% paraformaldehyde (Electron Microscopy Sciences, Hatfield, PA), dehydrated for 24 h in 30% sucrose (Sigma-Aldrich, St. Louis, MO), and then frozen in an embedding compound (Sakura Finetek, Tokyo, Japan) on dry ice. Next, 5-μm sections were rehydrated in 0.3% Triton X-100 (Phrmacia Biotech, Uppsala, Sweden) and blocked with 5% goat serum (abcam, Cambridge, UK) for 1 h. The sections were incubated with 2.5 μg/mL primary antibodies overnight at 4°C. We used the following anti-human primary mAbs: anti-Foxp3 (clone: 236A/E7), anti-CD4 (clone: EPR6855), and anti- CD103 (clone: EPR-4166); these mAbs were purchased from abcam (Cambridge, UK). Anti-Laminin Abs (clone: LAM-89) was purchased from Novus Biologicals (Littleton, Colorado, United States). After washing with 0.3% Triton X-100, the sections were incubated for 3 h at room temperature with the secondary antibodies. We used the following secondary antibodies: Alexa Fluor 488-conjugated goat anti-mouse IgG, Alexa Fluor 488-conjugated goat anti-rabbit IgG, Alexa Fluor 647-conjugated goat anti-mouse IgG, and Alexa Fluor 647-conjugated goat anti-rabbit IgG (1:500; Life technologies, Carlsbad, CA). The sections were mounted with VECTASHIELD Mounting Medium supplemented with DAPI (Vector Lab, Burlingame, CA). Human inflamed skin were fixed with 20% formalin, embedded in the paraffin, and then stained as is the case of frozen sections after deparaffinization and antigen retrieval with Target Retrieval Solution (pH6; Agilent Technologies, Santa Clara, California). Immunofluorescence images were obtained *via* fluorescence microscopy (BIOREVO BZ-9000; Keyence, Osaka, Japan).

### Quantitative PCR

Total RNA was extracted from human noninflamed and inflamed epidermis using QIAzol^®^ Lysis Reagent (Qiagen, Hilden, Germany) and RNeasy^®^ Plus Universal Mini kit (Qiagen, Hilden, Germany) per the manufacturer’s instructions. Reverse transcription was performed using ReverTra Ace^®^ qPCR RT Kit (Toyobo, Ohtsu, Japan) per the manufacturer’s instructions. mRNA levels were determined using commercially available primer/probe sets (TaqMan^®^ Gene Expression Assay: Applied Biosystems, Foster City, CA) and the AB7500 real-time PCR system (Applied Biosystems, Foster City, CA). The amount of target gene mRNA obtained using real-time PCR was normalized against the amount of housekeeping control gene (ACTB) mRNA. Human *Foxp3* primer was designed by Takara (Kyoto, Japan).

### T cell isolation

Dead cells were eliminated from the epidermal emigrants using Dead Cell Removal Kit (Miltenyi Biotec GmbH, Bergisch Gladbach, Germany). CD8^+^ T cells were positively isolated from the epidermal emigrants using CD8 microbeads (Miltenyi Biotec GmbH, Bergisch Gladbach, Germany). CD4^+^CD25^−^ and CD4^+^CD25^+^ T cells were isolated from both the epidermal emigrants and the PBMCs using CD4^+^CD25^+^ Regulatory T cell Isolation kit (Miltenyi Biotec GmbH, Bergisch Gladbach, Germany). To enhance the CD4^+^CD25^+^ T-cell purity, cells were passed through two-round consecutive columns. In the Treg suppression assay, isolated CD8^+^ T cells and CD4^+^CD25^−^ T cells that were used as responders were labeled with 2.5 μM CFSE (Invitrogen Life Technologies, Carlsbad, CA) for 10 min at 37°C. These responders were cocultured with or without isolated CD4^+^CD25^+^ T cells at 20:1 ratio in the 96-well round-bottom plates that were pre-immobilized with anti-CD3 (1 μg/mL; clone UCHT1; BioLegend, San Diego, CA) and anti-CD28 (3 μg/mL; clone CD28.2; BioLegend, San Diego, CA) mAbs overnight at 4°C. After culturing for 5 days at 37°C, the CFSE expression in the responders was determined by flow cytometry.

### Genomic DNA demethylation in the *FOXP3* gene

Isolated T cells were resuspended with 400 μL of Lysis buffer (100 mM NaCl, 100 mM Tris-HCL, 50 mM EDTA, 0.5% SDS). 2 μL of Proteinase K (20 mg/mL; Invitrogen, Waltham, MA) was added to the sample and incubated at 55 °C for 24 h at 1,300 rpm. 400 μL of PCI (phenol/chloroform/isoamyl alcohol (25:24:1)) were used for the phenol–chloroform extraction (twice). 400 μL of CIA (chloroform/isoamyl alcohol (24:1)) was added for the chloroform extraction. 40 μL of 3 M Sodium Acetate, 1 μL of ethatinmate (Nippon Gene, Tokyo, Japan) and 1 mL of 100% ethanol were used for the ethanol precipitation. The genomic DNA was eluted in 25 μL of TE buffer. Bisulfite treatment was performed using MethylEasy™ Xceed (Human Genetic Signatures, North Ryde, Australia) according to the manufacturer instructions. The target locus was amplified by TaKaRa Ex Taq^®^ Hot Start Version (Takara, Shiga, Japan) polymerase with bisulfite sequence primers previously reported (human *FOXP3* CNS2 forward primer: TTGGGTTAAGTTTGTTGTAGGATAG and reverse primer: ATCTAAACCCTATTATCACAACCCC) (17). The PCR product was subjected to gel electrophoresis and extracted using QIAEX II Gel Extraction Kit^®^ (Qiagen, Venlo, Netherland). TA cloning was performed using DynaExpress TA PCR Cloning kit (Funakoshi, Tokyo, Japan). DH5α transformation was performed using *E.coli* DH5α Competent Cells (Takara, Shiga, Japan) and incubated overnight at 37 °C. White colonies were picked up and the denature reaction and the rolling circle amplification were performed using illustra™ TempliPhi™ DNA Amplification Kit (Cytiva, Tokyo, Japan). Sequencing was performed using BigDye™ Terminator v3.1 (Applied Biosystems, Foster City, CA), M13 Reverse primer (Invitrogen Life Technologies, Carlsbad, CA) and 3500 Genetic Analyzer (Applied Biosystems, Foster City, CA).

### Statistical analysis

Statistical analysis was conducted using Student’s t test (one-tailed). Difference of *p < 0.05, **p < 0.01, and ***p < 0.001 were considered statistically significant.

## Results

### Higher IL-2Rα (CD25) and IL-2Rβ expressions in the epidermal CD4^+^CD103^−^ cells

**Figure 1:**
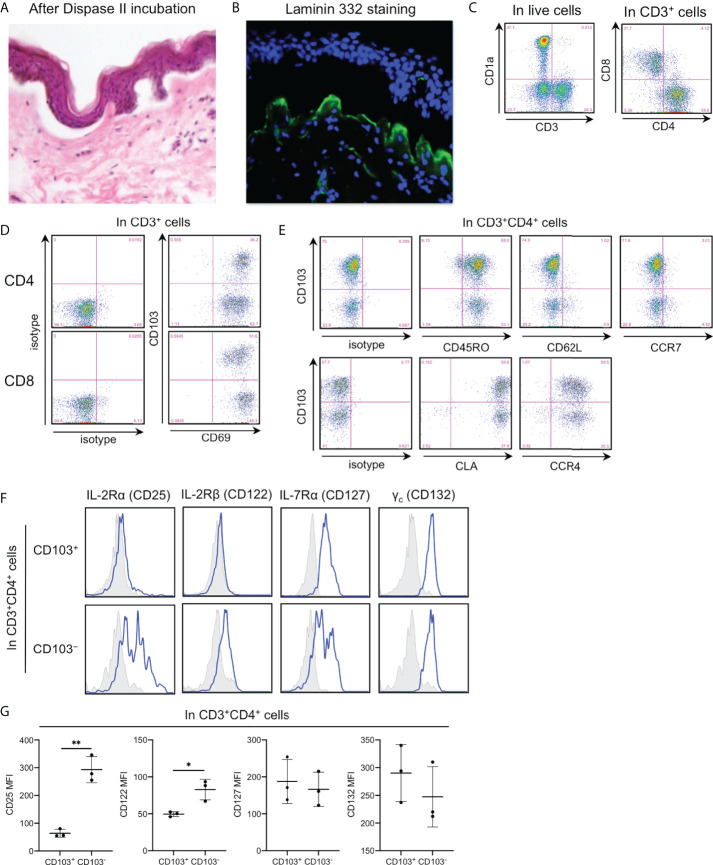
Higher IL-2Rα (CD25) and IL-2Rβ expressions in the epidermal CD4^+^CD103^−^ cells. **(A)** Hematoxylin–eosin staining of normal skin incubated with Dispase II overnight at 4°C. **(B)** Laminin 332 staining in **(A)**. **(C-F)** Representative expression of indicated antigens in indicated cell populations. **(G)** An MFI summary of **(F)**. All the experiments were conducted using at least three different samples. Comparisons between groups were evaluated using Student’s *t*-test (one-tailed); *p < 0.05 and **p < 0.01.

Unless otherwise noted, healthy and noninflamed human skin from female mamma was used in the experiments. For epidermal T cell analysis, the skin needs to be incubated with Dispase II to separate the epidermis from the underlying dermis (**Figure 1A**). Laminin 332, a major component of lamina densa, was stained in the dermal apical side (**Figure 1B**), indicating a lack of dermal components in the separated epidermis. In the spontaneous migration method, the epidermis was floated on a medium without adding any exogenous cytokines and stimulations for two days at 37°C (15). Then, emigrants from the epidermis were analyzed, followed by detecting CD1a^+^CD3^−^ LCs and CD1a^−^CD3^+^ T cells. The latter was divided into CD4^+^ and CD8^+^ T cells (**Figure 1C**), both of which expressed CD69, a T_RM_ marker, with or without CD103 (another T_RM_ marker) expression (**Figure 1D**), as previously reported (1, 15). Thereafter, epidermal CD4^+^ T cells were focused upon. Both the CD103^+^ and CD103^−^ fractions equivalently demonstrated a memory phenotype and skin tropism (**Figure 1E**). Common γ-chain and IL-7Rα expressions were comparable between CD103^+^ and CD103^−^ fractions, whereas IL-2Rα (CD25) and IL-2Rβ expressions were significantly higher in the CD103^−^ fraction (**Figures 1F, G**), suggesting higher IL-2 responsiveness.

### Predominant Foxp3 expression in the epidermal and epithelial CD4^+^CD103^−^ cells

**Figure 2:**
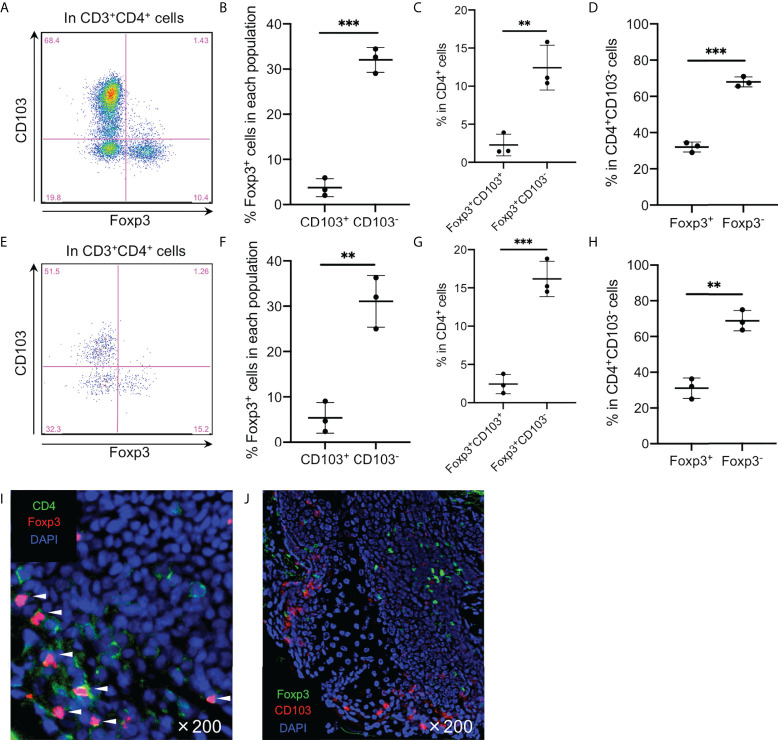
Predominant Foxp3 expression in the epidermal CD4^+^CD103^−^ cells. **(A, E)** Representative Foxp3 and CD103 expression in the CD3^+^CD4^+^ cells. **(B, F)** A summary of percentage of Foxp3^+^ cells in the epidermal CD103^+^ and CD103^−^ cells. **(C, G)** A summary of the indicated population percentage in the epidermal CD3^+^CD4^+^ cells. **(D, H)** A summary of the indicated population percentage in the epidermal CD3^+^CD4^+^CD103^−^ cells. **(A-D)** analyzed CD3^+^CD4^+^ cells of epidermal emigrants, whereas **(E-H)** analyzed CD3^+^CD4^+^ cells of enzymatically digested epidermal single-cell suspensions. **(I, J)** Representative CD4 and Foxp3 staining **(I)** and Foxp3 and CD103 staining **(J)** in normal skin. All the experiments were conducted using at least three different samples. Comparisons between groups were evaluated using Student’s *t*-test (one-tailed); **p < 0.01 and ***p < 0.001. Arrowheads in **(I)** indicate costained cells in the epidermis.

**Figure 3:**
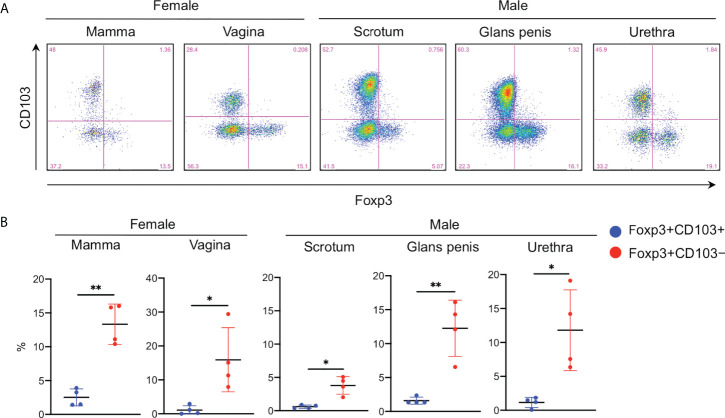
Predominant Foxp3 expression in the various epidermal and epithelial CD4^+^CD103^−^ cells. **(A)** Representative Foxp3 and CD103 expression in the epithelial CD3^+^CD4^+^ cells from the various organs. **(B)** A summary of the indicated population percentage in the epidermal CD3^+^CD4^+^ cells. One set of samples of mamma and vagina were not from same volunteers. This means these samples were from six different volunteers. Conversely, one set of samples of scrotum, gland penis, and urethra were from one volunteer. This means these samples were from three different volunteers. Comparisons between groups were evaluated using Student’s *t*-test (one-tailed); *p < 0.05 and **p < 0.01.

As expected in a high CD25 expression in the CD103^−^ fraction, Foxp3 was predominantly expressed in this fraction (**Figures 2A, B**). Additionally, Foxp3^+^CD103^−^ cells comprised ~15% of epidermal CD4^+^ T cells (**Figure 2C**) and approximately 40% of the CD103^−^ fraction consistently expressed Foxp3 (**Figure 2D**). However, the spontaneous migration method induces T cell activation (15) and Foxp3 is a T-cell activation marker (18). Thus, the epidermis was enzymatically digested with collagenase type IV, leading to a confirmed Foxp3 expression in the CD103^−^ fraction (**Figures 2E–H**), suggesting that Foxp3 expression in the CD103^−^ fraction was not induced by the spontaneous migration method. Consistent with flow cytometry-based data, the human epidermis from healthy volunteers contained small CD4^+^Foxp3^+^ cell populations (**Figure 2I**), and the expression of Foxp3 and CD103 was mutually exclusive (**Figure 2J**). Interestingly, Foxp3^+^CD103^−^ cells consistently comprised approximately 15% of epithelial CD4^+^ T cells in several female and male organs, except in the scrotal epidermis (**Figures 3A, B**). Based on a preferential localization of epidermal Tregs in the hair follicles (5, 6), hairy areas such as the scalp and face contain more Tregs (6). However, the scrotal epidermis, a hairy area, has fewer Tregs. In addition, epithelial Tregs existed consistently in tissues with LCs (e.g., skin (mamma) and type II mucosa (vagina and glans penis)) and without LCs (e.g., the type I mucosa (urethra)). Collectively, Foxp3 is expressed in CD103^−^ cells. In addition, epithelial Treg density might be different between parts of body. Moreover, epithelial Treg density does not only depend on hair density, and LCs are dispensable for epithelial Treg generation and maintenance.

### Tregs in the epidermis of inflammatory skin conditions

**Figure 4:**
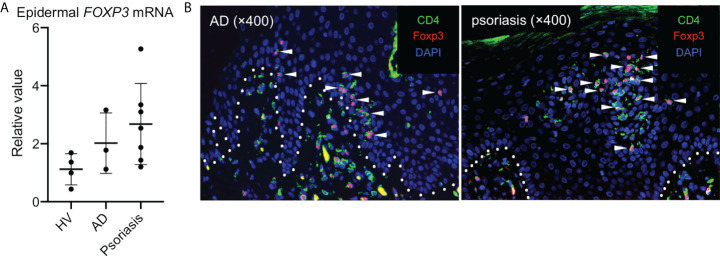
Tregs in the epidermis of inflammatory skin conditions. **(A)** A summary of *FOXP3* mRNA expression in the epidermis of indicated samples (HV: healthy volunteers and AD: atopic dermatitis). **(B)** Representative CD4 and Foxp3 staining in the skin of patients with AD (*left*) and with psoriasis (*right*). All the experiments were conducted using at least three different samples. Arrowheads in **(B)** indicate costained cells in the epidermis.

Tregs has been shown to increase the number and proliferating ability in the skin of patients with an inflammatory skin disease (e.g., psoriasis) (6). Thus, we aimed to determine whether Tregs increased specifically in the epidermis of patients with inflammatory skin diseases. To this end, epidermal *FOXP3* mRNA expression was examined using noninflamed mamma skin from healthy female volunteers and inflamed lesional trunk skin from male patients with treatment-naive atopic dermatitis and psoriasis. As a result, *FOXP3* mRNA expression (**Figure 4A**) and the number of Tregs marginally increased (**Figure 4B**). Therefore, Foxp3-expressing epidermal CD4^+^ T_RM_ cells increased in the epidermis of inflamed skin condition.

### Expression of Treg signatures in the epidermal Foxp3^+^CD4^+^ T cells and Foxp3^−^CD4^+^ T cells

**Figure 5:**
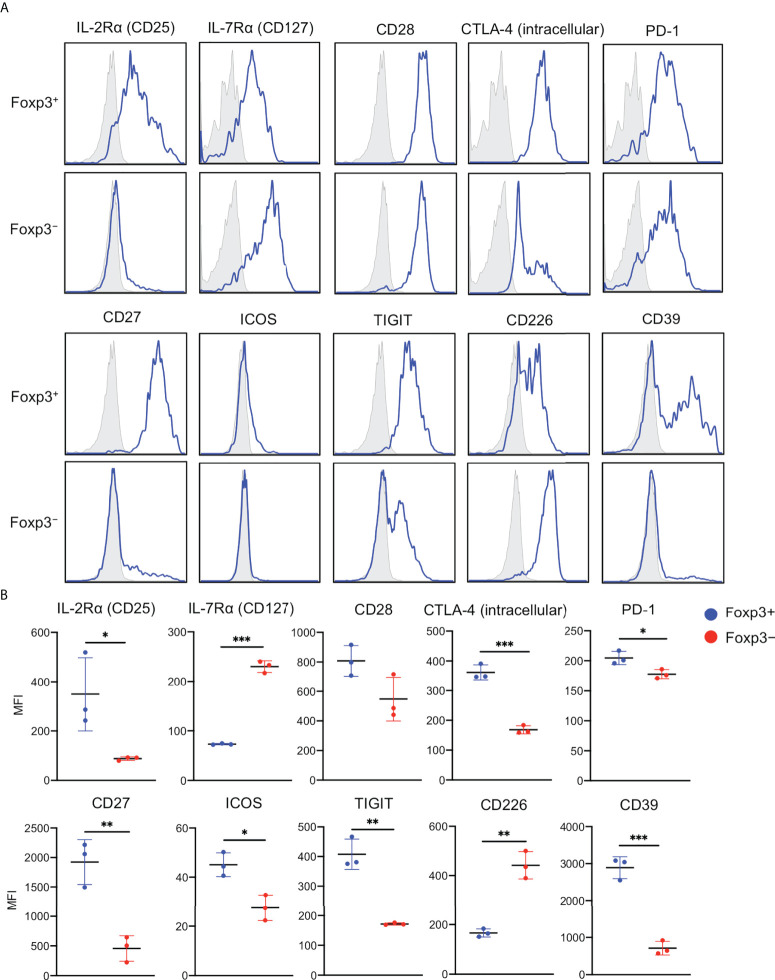
Expression of Treg signatures in the epidermal Foxp3^+^CD4^+^ T cells and Foxp3^−^CD4^+^ T cells. **(A)** Representative expression of indicated antigens in the epidermal Foxp3^+^ and Foxp3^−^ CD3^+^CD4^+^ cells. **(B)** An MFI summary of indicated antigens in the epidermal Foxp3^+^ and Foxp3^−^ CD3^+^CD4^+^ cells. All the experiments were conducted using at least three different samples. Comparisons between groups were evaluated using Student’s *t*-test (one-tailed); *p < 0.05, **p < 0.01, and ***p < 0.001.

In human CD4^+^CD25^+^Foxp3^+^ Tregs of peripheral blood and full-thickness skin, IL-7Rα (CD127) expression inversely correlates with suppressive function (6, 19). In addition, expression of Treg signatures like CTLA-4, PD-1, CD27, ICOS, TIGIT, and CD39 are associated with suppressive function and lineage stability. Thus, expressions of these molecules were examined. Epidermal Foxp3^+^CD4^+^ T cells expressed higher CD25 and lower CD127 than Foxp3^−^CD4^+^ cells. CD28 expression was comparable between both the fractions. However, the expressions of CTLA-4 (intracellular), PD-1, CD27, ICOS, TIGIT, and CD39 were significantly higher in Foxp3^+^CD4^+^ T cells than in Foxp3^−^CD4^+^ cells. In contrast, CD226 expression showed the opposite. Of note was that CD25, CD27, and CD39 were predominantly expressed in the Foxp3^+^CD4^+^ T cells (**Figures 5A, B**). These data indicate an expected suppressive capacity of epidermal Foxp3^+^CD4^+^ T cells.

### Less inflammatory cytokine production and activated phenotype of the epidermal Foxp3^+^CD4^+^ T cells

**Figure 6:**
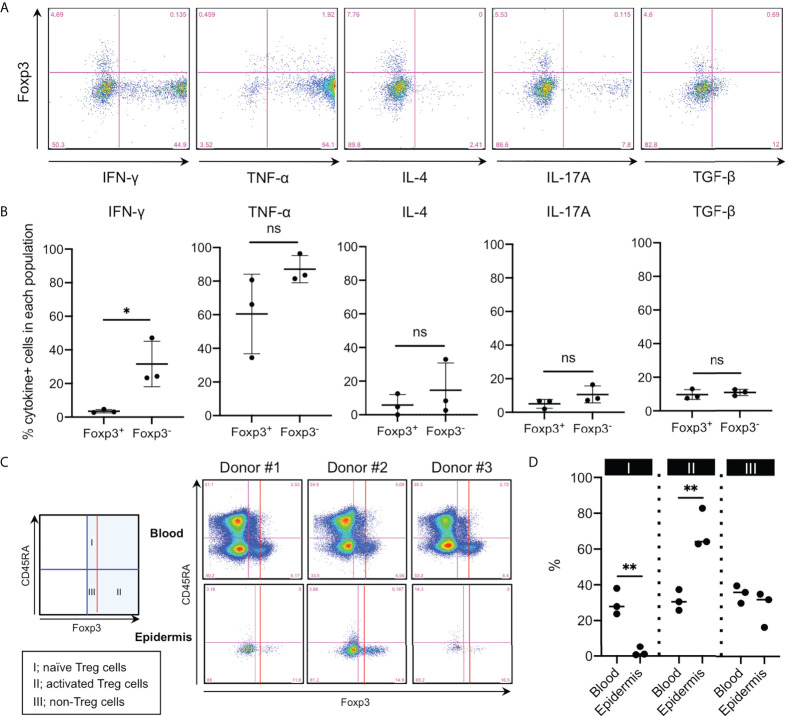
Less inflammatory cytokine production and activated phenotype of the epidermal Foxp3^+^CD4^+^ T cells. **(A)** Representative cytokine expression in the epidermal CD3^+^CD4^+^ cells. Cells were incubated with eBioscience Cell Stimulation Cocktail (500x) and eBioscience Protein Transport Inhibitor Cocktail (500x) for 6 h at 37°C. **(B)** A summary of cytokine-positive cells in the epidermal Foxp3^+^ and Foxp3^−^ CD3^+^CD4^+^ cells. **(C)** CD45RA and Foxp3 expression in CD3^+^CD4^+^ cells of PBMCs and the epidermis. **(D)** A summary of the three populations’ percentage in **(C)**. All the experiments were conducted using at least three different samples. Comparisons between groups were evaluated using Student’s *t*-test (one-tailed); *p < 0.05 and **p < 0.01. ns, not significant.

Human Tregs in the full-thickness skin reportedly produce less IFN-γ and IL-17A compared with non-Tregs (6). Thus, cytokine production by epidermal Foxp3^+^CD4^+^ T cells was examined. Epidermal Foxp3^+^CD4^+^ cells expressed significantly less IFN-γ and tended to express less TNF-α, IL-4, and IL-17A (**Figures 6A, B**), but showed comparable TGF-β expression, compared to those by epidermal Foxp3^−^CD4^+^ T cells (**Figures 6A, B**). IL-10 production was undetected regardless of Foxp3 expression in this system (data not shown).

Following CD45RA expression and Foxp3 intensity, Foxp3^+^ cells in the human peripheral blood are divided into the following three populations: Foxp3^+^CD45RA^+^ naive Tregs (fraction I), Foxp3^high^CD45RA^−^ activated Tregs (fraction II), and Foxp3^+^CD45RA^−^ non-Tregs (fraction III) (20). Thus, we sought to determine whether epidermal Foxp3^+^CD4^+^ T cells belonged to which fraction. Epidermal Foxp3^+^CD4^+^ T cells consisted of greater activated and less naive Tregs than peripheral blood Foxp3^+^CD4^+^ T cells (**Figures 6C, D**). These data suggest that epidermal Foxp3^+^CD4^+^ T cells are equipped with activated Treg phenotype.

### Highly demethylated *FOXP3* in the epidermal CD4^+^CD103^−^ cells

**Figure 7:**
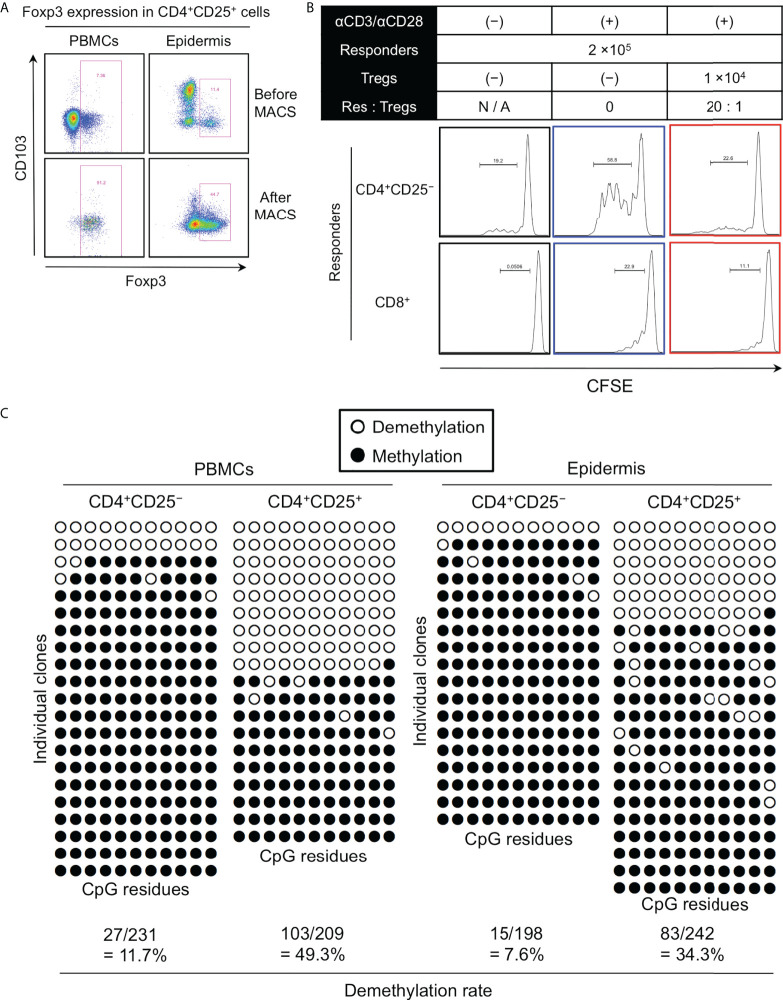
Highly demethylated *FOXP3* in the epidermal CD4^+^CD103^−^ cells. **(A)** Representative Foxp3 and CD103 expression in CD4^+^CD25^+^ cells of PBMCs and epidermal emigrants before and after magnetic sorting. **(B)** Representative CFSE expression in the indicated responders. **(C)** Representative genomic DNA demethylation in the *FOXP3* gene of CD4^+^CD25^−^ and CD4^+^CD25^+^ cells from PBMCs and the epidermis. All representative data were obtained from a single experiment due to the limited availability of huge skin samples that were required to conduct the experiments.

To validate the suppressive function of epidermal Foxp3^+^CD4^+^ T cells that mostly consisted of CD103^−^ fraction, the Treg population was magnetically enriched using the CD4^+^CD25^+^ Regulatory T cell Isolation Kit (Miltenyi Biotec). The Foxp3^+^ cell purity was enhanced by approximately 90% in the PBMCs but only up to 50% in the epidermis (**Figure 7A**). Nevertheless, this incompletely enriched epidermal Treg population strongly suppressed the proliferation of both allogeneic epidermal CD4^+^CD25^−^ T cells and CD8^+^ T cells (**Figure 7B**). Weak cytokine production and suppressive function by the peripheral blood Tregs strongly correlate with the genomic DNA demethylation in intron 1 of the *FOXP3* (TSDR locus) (20–22). Therefore, *FOXP3* in CD4^+^CD103^−^ Tregs with high CD25 expression is expected to be highly demethylated. Hence, the methylation patterns of upstream Foxp3 CpG islands in CD4^+^CD25^−^ and CD4^+^CD25^+^ T cells in the PBMCs and epidermis from female subjects were analyzed. The *FOXP3* demethylation rate was higher in CD4^+^CD25^+^ T cells of PBMCs and the epidermis than in CD4^+^CD25^−^ T cells (**Figure 7C**). As female samples were used in these experiments, Foxp3 in the epidermal CD4^+^CD25^+^ Tregs that mostly consisted of CD103^−^ cells appeared to be highly demethylated, as it is on the X-chromosome, resulting in X chromosome inactivation; thus the uppermost limit of *FOXP3* demethylation is 50% (23). Moreover, the Foxp3^+^ cell purity in the epidermal CD4^+^CD25^+^ yielded by CD4^+^CD25^+^ Regulatory T cell Isolation Kit was up to 50% (**Figure 7A**). These data suggest that the *FOXP3* gene in the epidermal CD4^+^CD25^+^CD103^−^ cells is highly demethylated and these epidermal Tregs have a potent suppressive function.

## Discussion

The presence of resident memory Tregs in human epidermis has been previously reported (5). They are abundant in hair follicle (5, 6). Furthermore, epidermal LCs regulate their proliferation (5). However, the precise phenotype is unknown. Therefore, the current study reported that Foxp3 was predominantly expressed in CD4^+^CD103^−^ T cells of several human epithelial types because *FOXP3* DNA in CD4^+^CD103^−^ T cells was highly demethylated compared to that in CD4^+^CD103^+^ T cells. Moreover, epidermal CD4^+^CD103^−^Foxp3^+^ cells from healthy and noninflamed human skin inhibited the proliferation of both allogeneic epidermal CD4^+^CD103^+^ and CD8^+^ T cells, which indicated a bona fide Treg identity. Foxp3-expressing epidermal CD4^+^ T_RM_ cells increased marginally in the epidermis of inflamed skin. However, given that Foxp3 is a T-cell activation marker (18), it was uncertain whether these cells were “true” Tregs.

Human epidermal and dermal T cells are divided into CD103^+^ and CD103^−^ cells (1, 15). Since CD103 (integrin αE) binds to E-cadherin expressed on keratinocytes (1, 24), CD103 on the T cells is likely required in tissue retention. However, the migratory behavior of human epidermal CD8^+^ T_RM_ cells was comparable irrespective of CD103 expression (25), suggesting that epidermal retention of human T cells is not mediated only by CD103. Human epidermal and dermal T_RM_ cells including Tregs express strongly a T_RM_ marker CD69 irrespective of CD103 expression (1, 15). CD69 has been shown to facilitate T-cell tissue retention (26, 27). Therefore, skin CD103^−^ T cells might keep their local retention *via* putative adhesion molecules (e.g., CD69).

Foxp3 was expressed predominantly in the CD4^+^CD103^−^ T cells of several human epithelial types. Consistently, human CD25^high^FoxP3^+^ Tregs barely express CD103 in various tissues such as blood (28). Conversely, Foxp3 is expressed preferentially in CD4^+^CD103^+^ T cells of murine skin and other murine organs and tissues (28). The underlying mechanisms by which CD103 is expressed differently by Foxp3^+^ Tregs in mice and humans remain unknown. Why is Foxp3 expressed in human CD103^−^ fraction? First, human thymic CD4 single-positive T cells are unable to express CD103 regardless of Foxp3 expression (29). The percentage of human skin Tregs in the CD4^+^ T cells reached a peak during the second trimester *via* both continued thymic egress and local proliferation (8). Moreover, *FOXP3* DNA in CD4^+^CD103^−^ T cells was demethylated significantly. These data suggest that the epidermal CD4^+^CD103^−^Foxp3^+^ cells examined in the current study are thymus-derived Tregs.

The CD103^−^ fraction of human epidermal CD4^+^ T_RM_ cells expressed strongly IL-2Rα (CD25) and IL-2Rβ. Additionally, Foxp3 was expressed predominantly in the CD103^−^ fraction. The data suggest that IL-2 is involved in the regulation of human epidermal Tregs. In the murine Tregs of secondary lymphoid organs and skin, IL-2 is indispensable in Treg generation (30), Foxp3 induction (31, 32), and stability of Foxp3 expression (33). Moreover, IL-2 induces the expression of CD25, CTLA-4, and CD39 in Tregs, thereby enhancing suppressive function (34). However, the maintenance of murine Tregs appears to be mediated by IL-7 rather than IL-2 (30, 32). Meanwhile, the regulation of human Tregs by common γ-chain cytokines is less known. In human peripheral blood and skin Tregs, IL-7Rα expression correlates inversely with suppressive function (6, 19). Additionally, the proliferation of human skin Tregs is mediated by IL-2 and IL-15 (5). Further analyses are required to elucidate the regulation of human Tregs by common γ-chain cytokines.

Furthermore, Treg signatures (e.g., CTLA-4, PD-1, CD27, ICOS, TIGIT, and CD39) were found predominantly in the CD4^+^Foxp3^+^ T cells. Murine Treg-specific CTLA-4 depletion leads to the spontaneous development of systemic lymphoproliferation and T-cell-mediated autoimmune diseases, and an impaired suppressive function (35). Conversely, murine Treg-specific PD-1 depletion leads to an enhanced activated phenotype and suppressive function (36). Both human and murine skin Tregs strongly express CD27, which inhibits Treg/Th17 plasticity (7). ICOS is dispensable in the induction of murine Foxp3; however, it labels Tregs with superior suppressive capacity (37) and promotes Treg survival (38). The coinhibitory and costimulatory factor of TGIT and CD226, respectively, bind with the common ligand CD155. TIGIT is upregulated on activated human peripheral blood Tregs, which facilitate lineage stability and suppressive capacity (39). CD39 is expressed primarily by immune-suppressive Tregs in both humans and mice; thereby, suppressing the development of inflammatory autoimmune diseases (40). These findings suggest that CD4^+^CD103^−^Foxp3^+^ cells play an important role in epithelial immune homeostasis. For example, depigmentation in vitiligo is mediated by IFN-γ-producing epidermal CD49a^+^CD8^+^ T_RM_ cells (41). We found that epidermal CD4^+^CD103^−^Foxp3^+^ cells inhibited the proliferation of both allogeneic epidermal CD4^+^CD103^+^ and CD8^+^ T cells. As a result, an immunological imbalance between CD4^+^CD103^−^Foxp3^+^ cells and other effector cells may initiate or worsen epidermal skin diseases. Furthermore, the abundance of T_RM_ cells in the human epidermis, including CD4^+^CD103^−^Foxp3^+^ cells, raises the possibility of autonomous immunity in the epidermis independent of the effect of dermal immunity.

As mentioned, epidermal Tregs are assumed to be regulated by LCs in the hair follicles. However, the reduced number of CD4^+^CD103^−^Foxp3^+^ cells in the scrotal epidermis, a hairy area, and the existence of CD4^+^CD103^−^Foxp3^+^ cells in the urethral epidermis devoid of hair follicles and LCs suggest that generation and maintenance of epithelial Tregs are independent of hair density and LCs. The underlying mechanisms by which epithelial Tregs are generated and maintained in such organs are left open.

There is no definitively superior method for analyzing both epidermal and dermal T cells whose antigen expression is not modulated, because Dispase II incubation alone can cleave T cell and T_RM_ markers (15). However, in terms of T cell antigen protection, the spontaneous migration method used in this study outperforms the enzymatic digestion method (15). Furthermore, we examined epidermal emigrants produced using the spontaneous migration method without the addition of any exogenous cytokines or stimulations. Thus, current data in this study originate from relatively natural human epidermal T cells.

A limitation in this study was the use of mamma skin from female volunteers. The information might vary if samples from male volunteers and/or other skin regions (e.g., scrotum) were used.

Lastly, further studies are needed to uncover the modulation of the phenotype and function of epithelial Tregs in the inflamed condition and the involvement of epithelial Tregs in the pathomechanisms of autoimmune skin diseases.

## Data availability statement

The data that support the findings of this study are available from the corresponding author, YO, upon reasonable request.

## Ethics statement

The studies involving human participants were reviewed and approved by The Institutional Review Board of the University Hospital. The patients/participants provided their written informed consent to participate in this study.

## Author contributions

YO conceived the project. YO, SS, AM, and TK designed the research. TS conducted all experiments with the helps of YO, YN, AI, IS, RW. KY and AT conducted *FOXP3* demethylation experiments. YO prepared the figures and wrote the manuscript. All other authors provided critical comments on the manuscript. All authors read and approved the final manuscript.

## Funding

YO received a grant from the Grants-in-Aid for Scientific Research (KAKENHI; grant no. 20K08687).

## Acknowledgments

We thank all the participants. We also thank Ms. Ogino for her technical assistance.

## Conflict of interest

The authors declare that the research was conducted in the absence of any commercial or financial relationships that could be construed as a potential conflict of interest.

## Publisher’s note

All claims expressed in this article are solely those of the authors and do not necessarily represent those of their affiliated organizations, or those of the publisher, the editors and the reviewers. Any product that may be evaluated in this article, or claim that may be made by its manufacturer, is not guaranteed or endorsed by the publisher.

## Abbreviations

IL, interleukin; TGF-β, transforming growth factor-β; MHC, major histocompatibility complex; mRNA, messenger ribonucleic acid; CTLA-4, cytotoxic T-lymphocyte-associated protein-4; PD-1, programmed cell death 1; ICOS, inducible T-cell co-stimulator; TIGIT, T cell immunoreceptor with Ig and ITIM domains; IFN, interferon; TNF, tumor necrosis factor; PBMCs, peripheral blood mononuclear cells; DNA, deoxyribonucleic acid; MFI, mean fluorescence intensity.
